# Improved Detection of *mecA*-Mediated β-Lactam Resistance in *Staphylococcus lugdunensis* Using a New Oxacillin Salt Agar Screen

**DOI:** 10.3389/fmicb.2021.704552

**Published:** 2021-08-06

**Authors:** Pak-Leung Ho, Ying-Hang Law, Melissa Chun-Jiao Liu, Andes Lau, Man-Ki Tong, Kin-Hung Chow, Alan Ka-Lun Wu, Cindy Wing-Sze Tse, Vincent Chi-Chung Cheng, Tak-Lun Que

**Affiliations:** ^1^Department of Microbiology, Queen Mary Hospital, University of Hong Kong, Hong Kong, China; ^2^Carol Yu Center for Infection, University of Hong Kong, Hong Kong, China; ^3^Department of Clinical Pathology, Tuen Mun Hospital, Hospital Authority, Hong Kong, China; ^4^Department of Clinical Pathology, Pamela Youde Nethersole Eastern Hospital, Hospital Authority, Hong Kong, China; ^5^Department of Clinical Pathology, Kwong Wah Hospital, Hospital Authority, Hong Kong, China

**Keywords:** cefoxitin, methicillin resistance and *mecA* gene, susceptibility test comparison, *Staphylococcus lugdunensis*, oxacillin agar screening method

## Abstract

Oxacillin resistance mediated by *mecA* in *Staphylococcus lugdunensis* is emerging in some geographic areas. We evaluated cefoxitin disk diffusion (DD) and a new oxacillin agar (supplemented with 2 μg/ml oxacillin and 2% sodium chloride) screen for the detection of *mecA*-mediated resistance in *S. lugdunensis*. A total of 300 consecutive, non-duplicated clinical *S. lugdunensis* isolates from diverse sources in Hong Kong in 2019 were tested. The categorical agreement and errors obtained between cefoxitin DD test, oxacillin agar screen and *mecA* PCR were analyzed. Isolates with discordant results were further tested by MIC, penicillin binding protein 2a (PBP2a) assays, population analysis and molecular typing. PCR showed that 62 isolates were *mecA*-positive and 238 isolates were *mecA*-negative. For cefoxitin DD results interpreted using *S. aureus*/*S. lugdunensis* breakpoints, the categorical agreement (CA) for two brands of Muller-Hinton agars, MH-II (Becton Dickinson) and MH-E (bioMérieux) were both 96.0%; MEs were both 0%; and VMEs were 19.4 and 12.9%, respectively. The new oxacillin agar reliably differentiated *mecA*-positive and *mecA*-negative isolates (100% CA) without any ME or VME results. The 8 isolates with false susceptibility in the cefoxitin DD testing had cefoxitin and oxacillin MICs in the susceptible range. The isolates showed heterogeneous oxacillin resistance with resistant subpopulations at low frequencies. All had positive PBP2a results and were typed as sequence type 27/SCC*mec* V. The findings highlight the inability of cefoxitin DD and MIC tests for reliable detection of some *mecA*-positive *S. lugdunensis* isolates.

## Introduction

*Staphylococcus lugdunensis* is an unusual coagulase-negative *Staphylococcus*. Although coagulase-negative staphylococci are relatively avirulent in immunocompetent host, *S. lugdunensis* is an exception (Heilbronner and Foster, 2021). Bacteraemia is frequently accompanied by endocarditis, involving native valve more frequently than prosthetic valve, and it runs a clinical course resembling *S. aureus* infection (Kyaw et al., 2018). It has also been reported to cause other invasive infections such as brain abscess, osteomyelitis, septic arthritis and peritonitis (Heilbronner and Foster, 2021). The organism colonizes the groin, perineum and rectum, and has been associated with many infections over these areas (Bieber and Kahlmeter, 2010). Antibiotic treatment of staphylococcal infection can be impeded by the acquisition of resistance to multiple classes of antibiotics, especially β-lactams. In staphylococci, β-lactam resistance is mostly mediated by *mecA*, encoding PBP2a, which catalyzes cell wall synthesis in the presence of otherwise inhibitory concentrations of beta-lactam. Since oxacillin or cefoxitin is used as a surrogate for phenotypic detection *mecA*-mediated resistance, isolates that contain *mecA* are called oxacillin (or cefoxitin)-resistant, while isolates lacking *mecA* are designated oxacillin (or cefoxitin)-susceptible (CLSI, 2020).

In staphylococci, the expression of *mecA* is regulated by genes within (*mecI*, *mecR*) and outside (global regulator genes, SOS response genes, guanine metabolism genes) the SCC*mec* element (El-Halfawy and Valvano, 2015; Pardos et al., 2018). The diversity of the SCC*mec* elements and the bacterial host species may also affect the *mecA*-mediated resistance phenotypes (Chen et al., 2017). This is partly reflected in the different choices of test methods (MIC or disc), test drug (cefoxitin, oxacillin), and breakpoints recommended for the detection of *mecA*-mediated resistance in different *Staphylococcus* species (CLSI, 2020). Although the Clinical and Laboratory Standards Institute (CLSI) currently recommends the same set of test methods and breakpoints for *S. aureus* and *S. lugdunensis*, the reliability of the recommendations has not been validated using a large collection of *mecA*-positive *S. lugdunensis* (CLSI, 2020). Recently, we showed that oxacillin resistance in some *mecA*-positive *S. lugdunensis* isolates cannot be reliably detected by the current disc (cefoxitin) and MIC (cefoxitin and oxacillin) breakpoints, and that improved detection can be achieved using a new oxacillin-salt agar (Muller-Hinton [MH] supplemented with 2 μg/ml oxacillin and 2% sodium chloride) (Ho et al., 2020). In the current work, we assessed the diagnostic performance of the new oxacillin-salt agar for detection of oxacillin resistance in consecutive, clinical *S. lugdunensis* isolates. An algorithm involving the combined use of oxacillin salt agar screen, cefoxitin disc and rapid PBP2a detection for routine use in clinical laboratory is proposed.

## Materials and Methods

### Study Design and Bacterial Identification

Three public hospital laboratories (K, Q, and T) located in three healthcare regions in Hong Kong participated in this study. The laboratories were in diverse geographic areas and were estimated to provide testing for one third of the Hong Kong population. Each healthcare region has a cluster of hospitals that provides a comprehensive range of inpatient service including accidents and emergency department, intensive care unit, pediatrics, obstetrics and gynecology. The healthcare region supported by laboratory Q includes a University-affiliated hospital with a full range of stem cell and solid organ transplantations. From January to September 2019, each laboratory was requested to submit 100 consecutive, single-patient isolate of *S. lugdunensis*. Patient identities were kept anonymous. The following information was provided by the submitting laboratories: sex, age, date of collection, specimen source and patient location. Only isolates recovered from clinical specimens were included. Isolates that failed to grow or grow poorly on Muller-Hinton agars were excluded from the cefoxitin disc analysis (Supplementary Figure 1). Only one isolate per patient was included. The isolates were stored in MicroBank (Pro-Lab Diagnostics, Neston, United Kingdom) at −80°C and were sub-cultured twice on 5% blood agar before testing. All the isolates were retested for this work by one person who is a registered medical laboratory technologist with 2 years of full-time working experience in clinical microbiology. The isolates were identified by MALDI-TOF MS using a Bruker microflex LT system (Bruker Daltonics, Bremen, German) using a reference library of 8326 standard spectra (DB-8326 MSP).

### Molecular Studies

A multiplex PCR was used to detect the *mecA* and *mecC* genes in all the isolates (Ho et al., 2020). Multilocus sequence typing and SCC*mec* types were determined by next generation sequencing using an Illumina NovaSeq Platform at an output of approximately 1 Gb per isolate, as previously reported (Ho et al., 2016; Liu et al., 2019). The Pasteur database^1^ was used for assignment of sequence type (ST). The combination of *ccr* gene complex and *mec* gene complex was used to assign SCC*mec* types (International Working Group on the Classification of Staphylococcal Cassette Chromosome Elements [IWG-SCC], 2009). Details on the bioinformatics methods have been previously published (Ho et al., 2016; Liu et al., 2019).

### Detection of *mecA* Positive Isolates by Cefoxitin Disc Diffusion Test and Screening With Oxacillin Agar

Cefoxitin disc diffusion (DD) tests were performed as described by the CLSI using Muller-Hinton E (MH-E) agar (bioMérieux, Marcy-l’Étoile, France) and Muller-Hinton II (MH-II) agar from Becton Dickinson (BD, Hong Kong). The ability of a new oxacillin agar screening for detection of *mecA*-mediated resistance in *S. lugdunensis* was assessed following the CLSI’s procedure (CLSI, 2020). An in-house Muller-Hinton II (BD, Hong Kong) agar supplemented with 2% sodium chloride and 2 μg/ml oxacillin was used. Bacteria were cultured on 5% horse blood agar plates overnight. At least five colonies were sampled and a 0.5 McFarland standard bacterial suspension was prepared by the colony suspension method. A 5 μL disposable loop was dipped into the suspension and each agar was spot inoculated an area 10–15 mm in diameter. The inoculated plates were incubated at 35°C in ambient air and examined after 24 h. Absence and presence of growth (>1 colony or thin film) was interpreted as indicating susceptibility and resistance, respectively. Cefoxitin DD test results were interpreted according to the CLSI (inhibition zone diameter, susceptible ≥22 mm, resistant, ≤21 mm) (CLSI, 2020). All the 300 isolates were tested by DD tests and oxacillin agar screen. On each day of testing, *S. aureus* ATCC 25923, ATCC 29213 and ATCC 43300 were included for quality control.

### PBP2a Test

The *mecA*-positive isolates that were cefoxitin disc susceptible in both MH-II and MH-E agars were investigated further for PBP2a expression using the MRSA-Screen test (Denka Seiken Co., Ltd., Japan). Colonies grown on 5% blood agar for 24 h were tested according to the manufacturer’s instructions and controls (*S. aureus* ATCC 43300 and *S. aureus* ATCC 25923) included in each run.

### Discrepant Result Resolution

The subset of isolates that gave discrepant results with *mecA* PCR were further tested by MIC determination using Sensititre plates (Thermo Fisher Scientific, United Kingdom) containing cation-adjusted Muller-Hinton broth with cefoxitin (unsupplemented, cefoxitin 4 and 8 μg/ml) and oxacillin (supplemented with 2% sodium chloride, oxacillin 0.25, 0.5, 1, 2, and 4 μg/ml). In each run, *S. aureus* ATCC 25923 and ATCC 29213 were included for quality control. Additionally, cefoxitin DD tests were repeated using MH-II and MH-E agars.

### Population Analysis

Analysis of oxacillin-resistant subpopulations was carried out as using a microdilution plating method (Pfeltz et al., 2001). Bacteria were subcultured onto blood agar plates and incubated overnight at 35°C. A bacterial suspension with a turbidity of 0.6 ± 0.02 optical density at 600 nm (equivalent to ∼10^9^ colony forming units/ml, CFU/ml) was prepared by sampling 5–10 colonies from the blood agar plate. The suspension was serially diluted (10^–1^–10^–7^) and from each five 10 μl droplets were plated onto MH-II agars containing a range of oxacillin concentrations (0, 1, 2, 4 μg/ml) (Pfeltz et al., 2001). The colonies were counted after 48 h of growth at 35°C. *Staphylococcus lugdunensis* ATCC 700328 (*mecA*-negative) and a *S. lugdunensis* strain K-070 (*mecA*-positive, oxacillin MIC ≥ 8 μg/ml, cefoxitin MIC 8 μg/ml, confluent growth pattern in oxacillin agar screen) were included as controls. Experiments were performed twice.

### Effect of Passage on Oxacillin-Containing and Antibiotic-Free Agars on the Oxacillin Resistance Phenotype

The effect of serial passage on oxacillin-containing and antibiotic-free agar on the oxacillin resistance phenotype was further investigated in two of the cefoxitin-susceptible/*mecA*-positive strains (K-016 and Q-015). First, the strains were cultured on antibiotic-free blood agar at 35°C overnight. Population analysis was performed using colonies from the blood agar plate (first generation). One discrete colony from the blood agar plate was picked and subcultured onto MH agar supplemented with 2 μg/ml oxacillin (quadrant streaking for isolation). After overnight incubation, colonies from the oxacillin agar plate (second generation growth) were picked for repeating the population analysis as described above. One discrete colony from the oxacillin-containing MH agar were then subcultured to antibiotic-free blood agar plates (quadrant streaking for isolation), and the plates were incubated at 35°C overnight. Afterward, one discrete colony from the blood agar plate (third generation growth) was picked for repeating the population analysis. Serial daily subculture onto blood agar plate was repeated daily and testing performed for a total of 7 days to assess the stability of the resistance phenotype.

### Data Analysis

Disc results were interpreted using the CLSI M100-2020 *S. aureus*/*S. lugdunensis* cefoxitin breakpoint (resistant, ≤21 mm). The *mecA* PCR result was used as the “gold standard” and compared against the cefoxitin disc test or agar screen results. Categorical agreement (CA), major error (MEs), and very major error (VMEs) were calculated as previously described (Huse et al., 2018). MEs were defined by isolates phenotypically resistant but *mecA*-negative. VMEs were defined by isolates phenotypically susceptible but *mecA*-positive. The ME and VME rates were calculated by using the total number of *mecA*-negative isolates and *mecA*-positive isolates as denominators, respectively. Previously proposed inter-method error rate of ≤ 3% (lower 95% confidence interval ≤ 1.5% and upper 95% confidence interval ≤ 7.5%) for VME was considered to be acceptable (Food and Drug Administration, 2007).

### Statistical Analysis

Proportions were compared using Chi-squared test. Continuous variables were tested by the paired sample *t-*test. Graph Prism and SPSS statistical packages were used to perform the statistical analyses. A two-tailed *P*-value of < 0.05 was considered as significant. The sample size in this study was chosen according to the FDA’s recommendation of 3 laboratories and 100 isolates per laboratory (Food and Drug Administration, 2007). Based on the final number of 62 resistant (*mecA*-positive) isolates; there should be zero number of VME for a method to be acceptable (Food and Drug Administration, 2007).

## Results

### Sources of the Isolates and Prevalence of *mecA*

During the study periods, a total of 310 isolates were submitted for analysis (Supplementary Figure 1). Ten isolates grew poorly on one (*n* = 1, MH-E) or both (*n* = 9) of the MH agars and were excluded. Therefore, 300 isolates (100 from each laboratory) were included in the DD testing and oxacillin agar screen. The majority of the isolates originated from wounds (94.6%, *n* = 284) including wound tissues (*n* = 24), pus (*n* = 104), aspirate (*n* = 3) and miscellaneous wound swabs (*n* = 153) (Supplementary Table 1). The remaining isolates were from various specimen types, including urine (*n* = 6), blood (*n* = 4), joint fluid (*n* = 1), respiratory (*n* = 3), and stool (*n* = 2). The two stool isolates were obtained from stem cell transplant recipients during neutropenic sepsis. The specimens were collected from patients in the accidents and emergency departments (21%, *n* = 63), outpatient clinics (7.7%, *n* = 23) and various inpatient departments (71.3%, *n* = 214). All isolates were identified as *S. lugdunensis* by MALDI-TOF at score of ≥ 2.0. Overall 20.7% (62/300) isolates were *mecA*-positive (Supplementary Table 1). The proportion of *mecA*-positive isolates in the laboratory Q (30%) was significantly higher than that in laboratory K (17%, *P* = 0.04) and T (15%, *P* = 0.01). All isolates were *mecC*-negative.

### Results by Cefoxitin Disc Diffusion Test and Oxacillin Agar Screen

**FIGURE 1:**
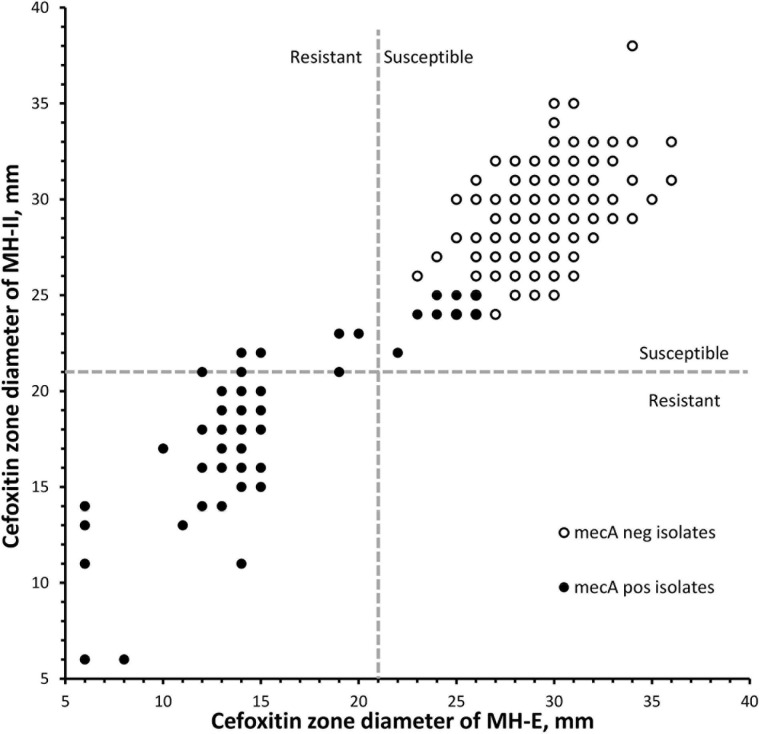
Scatterplot of cefoxitin inhibition zone diameters of 300 *Staphylococcus lugdunensis* isolates using two brands of Muller-Hinton agars. Dotted lines indicated the cefoxitin breakpoints for *S. aureus*/*S. lugdunensis* according to the CLSI M100-2020. MH-E, Muller-Hinton E agar from bioMérieux and MH-II, Muller-Hinton II agar from Becton Dickinson.

Results from the DD testing and oxacillin agar screen were summarized in Figure 1 and Table 1. Zones of inhibition in MH-II agar were generally larger than those in MH-E agar, especially for the *mecA*-positive isolates (Figure 1). The mean zones of inhibition in MH-II and MH-E for the *mecA*-positive isolates were 17.7 ± 4.6 mm and 14.2 ± 5.0 mm, respectively (*P* < 0.001). Those for *mecA*-negative isolates were 28.8 ± 2.2 mm and 29.3 ± 2.2 mm, respectively (*P* = 0.001). On applying the CLSI breakpoint for DD testing using cefoxitin, the CA values for MH-II and MH-E were both 96.0%; MEs were both 0%; and VMEs were 19.4 and 12.9%, respectively (Table 1). The difference in the VME rates for the two brands of MH media were not statistically significant (Fisher Exact test, *P* = 0.465). Four *mecA*-positive isolates were cefoxitin-susceptible in MH-II agar (zone sizes, 22–23 mm; repeated testing 22–27 mm) but cefoxitin-resistant in MH-E agar (zone sizes 14–19 mm; repeated testing 15–20 mm). An additional four isolates were cefoxitin-resistant in both agars but the zone sizes in MH-E agar (range, 12–19 mm) were smaller than in MH-II agar (all were 21 mm) (Figure 1). Another eight *mecA*-positive isolates were cefoxitin-susceptible in both MH-II (zone sizes 22–25 mm; repeated testing 23–26 mm) and MH-E (zone sizes 22–26 mm; repeated testing 22–25 mm) agars. Screening with oxacillin agar showed 100% CA for all 300 isolates with no ME or VME (Table 1).

**TABLE 1:** Performance of cefoxitin DD and oxacillin agar screen for detecting of *mecA*-mediated oxacillin resistance in *S. lugdunensis*.

	**No. of *mecA***		**% (95% CI)**		
	**Neg**	**Pos**	**CA**	**ME**	**VME**
**MH-II agar**					
Cefoxitin-R	0	50	96.0 (93.1–97.7)	0 (0–1.6)	19.4 (11.4–30.9)
Cefoxitin-S	238	12			
**MH-E**					
Cefoxitin-R	0	54	96.0 (93.1–97.7)	0 (0–1.6)	12.9 (6.7–23.5)
Cefoxitin-S	238	8			
**Oxacillin salt agar screen^#^**					
Growth	0	62	100.0 (98.4–100.0)	0 (0–1.6)	0 (0–7.3)
No growth	238	0			

*% for CA, category agreement; ME, major error; VME, very major error.*

*^#^Muller-Hinton agar supplemented with 2% sodium chloride and 2 μg/ml oxacillin.*

**FIGURE 2:**
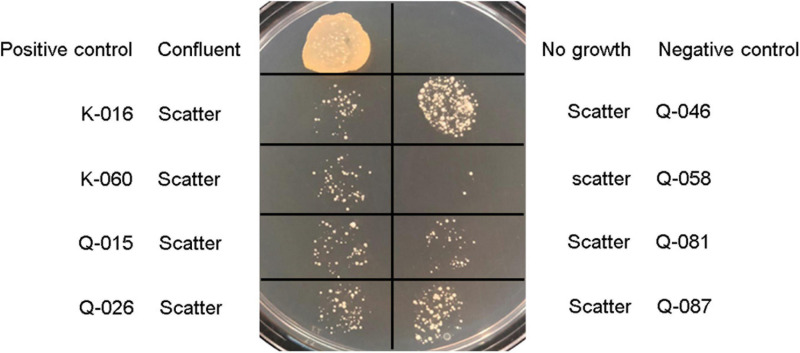
Growth patterns of *Staphylococcus lugdunensis* strains and *S. aureus* controls on Muller-Hinton agar supplemented with 2% sodium chloride and 2 μg/ml oxacillin using bacterial inoculum of 5 μl: Positive control, *S. aureus* ATCC 43300; negative control, *S. aureus* ATCC 29213; and *mecA*-positive, cefoxitin-susceptible *S. lugdunensis* strains (K-016, K-060, Q-015, Q-026, Q-046, Q-058, Q-081, and Q-087).

In the oxacillin agar screen, two different growth patterns were observed. Isolates in the cefoxitin DD-resistant/*mecA*-positive (*n* = 54) and cefoxitin DD-susceptible/*mecA*-positive (*n* = 8) group all yielded confluent and scatter growth patterns, respectively (Figure 2). The cefoxitin DD-susceptible/*mecA*-positive isolates were collected from 3 children and five adults; all from wounds (Table 2). Two were from hospital K and six were from hospital Q. The specialties of the eight patients were diverse (3 pediatrics, 2 accidents and emergency department, 1 medical, 1 obstetrics and gynecology, and 1 bone marrow transplantation). All isolates were of ST27 and SCC*mec* V. The 8 cefoxitin DD-susceptible/*mecA*-positive isolates had cefoxitin and oxacillin of MIC ≤ 4 μg/ml and 1–2 μg/ml, respectively. All the isolates were interpreted as cefoxitin- and oxacillin-susceptible by the MIC tests. Cefoxitin zone diameters for the two brands of Muller-Hinton agars were highly consistent. In the PBP2a test, all 8 isolates showed positive results, confirming *mecA* expression.

**TABLE 2:** Summary of 8 *mecA* -positive interpreted as cefoxitin-susceptible by the CLSI M100, 30th edition *S. aureus/S. lugdunensis* breakpoint.

**Strain**	**Date collected**	**Source**	**Specialty**	**Sex/age**	**Source**	**MLST/SCC*mec***	**Zone diameter, mm^a^**	**MIC, μ g/ml^b^**		**PBP2a**
							**Cefoxitin**	**Cefoxitin**	**Oxacillin**	
K-016	10-Feb-19	Hosp K	AED	M/30 year	Wound	ST27/SCC*mec* V	23/24 (S/S)	≤4 (S)	2	+
K-060	24-May-19	Hosp K	Medical	M/20 year	Wound	ST27/SCC*mec* V	25/24 (S/S)	≤4 (S)	1	+
Q-015	12-Mar-19	Hosp Q	Pediatrics	F/16 year	Wound	ST27/SCC*mec* V	26/25 (S/S)	≤4 (S)	1	+
Q-026	27-Mar-19	Hosp Q	BMT	M/47 year	Wound	ST27/SCC*mec* V	26/24 (S/S)	≤4 (S)	2	+
Q-046	21-May-19	Hosp Q	Pediatrics	M/1 day	Wound	ST27/SCC*mec* V	22/22 (S/S)	≤4 (S)	2	+
Q-058	23-May-19	Hosp Q	Pediatrics	M/2 month	Wound	ST27/SCC*mec* V	24/25 (S/S)	≤4 (S)	1	+
Q-081	25-Jun-19	Hosp Q	O&G	F/38 year	Wound	ST27/SCC*mec* V	25/25 (S/S)	≤4 (S)	1	+
Q-087	28-Jun-19	Hosp Q	AED	M/32 year	Wound	ST27/SCC*mec* V	24/24 (S/S)	≤4 (S)	1	+

*Hosp, hospital; AED, accidents and emergency department; BMT, bone marrow transplantation; O&G, obstetrics and gynecology; MLST, multilocus sequence typing; ST, sequence type; S, susceptible; R, resistant; PBP2a, penicillin-binding protein 2a; +, positive.*

*^*a*^Disc diffusion results obtained using MH-E and MH-II agars were given before and after /, respectively.*

*^*b*^The results were interpreted using the CLSI M100-2020 S. aureus/S. lugdunensis MIC (cefoxitin, S ≤ 4 μg/ml, R ≥ 8 μg/ml; oxacillin S ≤ 2 μg/ml, R ≥ 4 μg/ml) and disc breakpoints (cefoxitin zone diameter, S ≥ 22 mm, R, ≤ 21 mm) (CLSI, 2020).*

### Analysis of Oxacillin-Resistant Subpopulations

**FIGURE 3:**
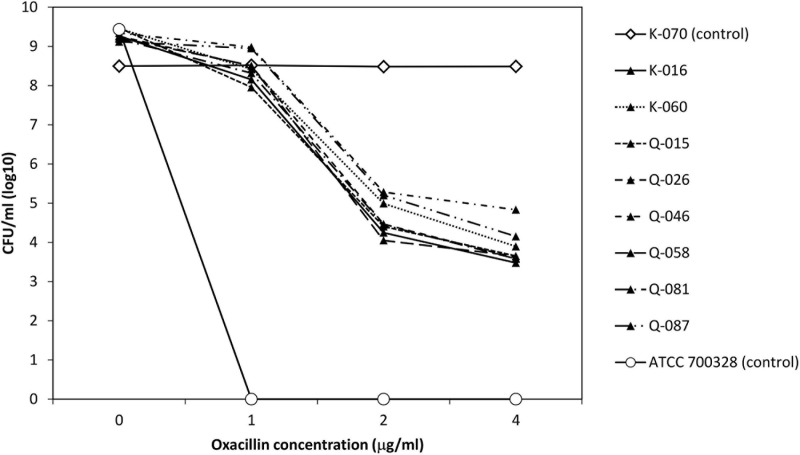
Analysis of oxacillin-resistant subpopulations of 8 cefoxitin DD-susceptible/*mecA*-positive strains and two control strains.

To evaluate the apparent β-lactam susceptibility in more details, we performed population analysis using oxacillin (Figure 3). In the 8 strains, oxacillin-resistant subpopulations (≥4 μg/ml) occurred at frequencies of one in 10^4^–10^5^. Thus, the apparent oxacillin susceptibility was interpreted as a result of the heterogeneous resistance expression. In contrast, the control strain showed homogeneous oxacillin-resistant phenotype.

### Effect of Passage on Oxacillin-Containing and Antibiotic Free Agars on the Oxacillin Resistance Phenotype

Two strains (K-016 and Q-015), one each from each hospital source were randomly selected for this part of the experiments. Both isolates expressed heteroresistance to oxacillin in the original testing at frequencies of ∼10^–6^ (Figure 3). Colonies that grew on the oxacillin agar plate had homogeneous oxacillin resistance by population analysis. In both strains, the homogeneous oxacillin-resistant phenotype was stably maintained following daily, serial passage in antibiotic-free agars for 7 days.

### Findings for Isolates With Poor Growth on MH Agars

The 10 isolates that show poor growth in the two MH agars were further tested. One of the ten isolates was *mecA*-positive. In the PBP2a test, the *mecA*-positive isolate had positive result. The cefoxitin and oxacillin MIC of this isolate was ≥16 μg/ml (resistant) and ≥8 μg/ml (resistant), respectively. The other 9 *mecA*-negative isolates had oxacillin MIC of ≤ 0.25–2 μg/ml (all susceptible) and cefoxitin MIC of ≤ 4 μg/ml (all susceptible).

## Discussion

We presented cefoxitin DD data for detection of *mecA*-mediated oxacillin resistance in 300 clinical isolates of *S. lugdunensis*. PCR for *mecA* was used to define oxacillin resistance. Our data showed that cefoxitin DD testing does not accurately predict the presence of *mecA* in *S. lugdunensis* by the use of the M100-2020 breakpoints for *S. aureus/S. lugdunensis*. The findings are consistent with previous studies that have shown the accuracy of cefoxitin DD testing in staphylococci to be species-dependent (Ferreira et al., 2012; Johnson et al., 2014; Wu et al., 2016; Huse et al., 2018; Morris and Simner, 2019). In this study, the accuracy (category agreement) of the cefoxitin DD test was 96%. A similar accuracy of cefoxitin DD test was found in another study that investigated 117 *S. lugdunensis* isolates from Taiwan hospitals (Kao et al., 2021) *mecC* was not detected in our bacterial collection, confirming *mecA* as the dominant mechanism of β-lactam resistance in *S. lugdunensis*.

Two widely used brands of MH agars were evaluated for their utility in detecting *mecA*-positive *S. lugdunensis*. Twelve and eight isolates were falsely susceptible using MH-II and MH-E agars, respectively (Table 1). The MH-II agar plates were prepared in-house using commercial powders in the testing laboratory while MH-E agar plates were factory made. Given that results for the quality control strains were all within range, the influence of in-house preparation on the observed results is likely to be minimal. Commercial MH media are recognized to have medium-to-medium variability of cation or other components (Girardello et al., 2012). In *S. schleiferi*, the VME rates have been reported to range 69–76% for three brands of MH agars (Huse et al., 2018). In a study that evaluated the ability of cefoxitin DD testing for detection of *mecC*-positive *S. aureus*, a former MH agar from bioMerieux had lower sensitivity than MH agars from Oxoid or BD (Skov et al., 2014). Our better results using MH-E is in agreement with the improved performance of the MH-E over the former MH agar in the detection of both *mecA* and *mecC* in *S. aureus* (Larsen et al., 2015).

Population analysis showed that the eight cefoxitin DD-susceptible/*mecA*-positive strains displayed the heterogeneous resistance phenotype in which there is a mixture of cell subpopulations with different level of oxacillin resistance. This explained why the eight strains yield a scatter growth patterns in the oxacillin agar screen (Figure 2). Since resistant subpopulations may occur at a low frequency, a sufficient number of cells should be spread onto the oxacillin agar plate. The CLSI recommended using a 1-μL loop for spreading a bacterial suspension of 0.5 McFarland turbidity onto oxacillin agar salt agar as an option for detection of *mecA*-mediated resistance in *S. aureus* (CLSI, 2020). This and our previous work showed that the use of a higher inoculum (5-μL loop) is essential for reliable detection of the cefoxitin-DD susceptible/*mecA*-positive *S. lugdunensis* isolates (Ho et al., 2020). In this study, sodium chloride was added to the test medium to enhance *mecA* expression and the 2% concentration follows that recommended for oxacillin MIC determination using the agar dilution method (Huang et al., 1993). Previous studies have shown that many *mecA*-negative *S. lugdunensis* isolates have an oxacillin MIC of 0.5–1 μg/ml (Frank et al., 2008). Therefore, the screening agar used oxacillin at 2 μg/ml. Findings from the population analysis suggested that oxacillin concentration over the 2–4 μg/ml range is likely to perform similarly (Figure 3). Colonies on the oxacillin agar plate were entirely resistant (i.e., homogeneous resistance) and this phenotype remained stable after repeated, serial passage on antibiotic free medium. Using mathematical modeling, it has been showed that this type of heteroresistance can result in the failure of antibiotic treatment of infections with bacteria that are misclassified as antibiotic susceptible (Nicoloff et al., 2019).

Some of the *S. lugdunensis* isolates did not grow on MH agars. In the past, it was common for laboratories to perform susceptibility on blood MH agar that supported growth of these variant isolates. However, the use of blood MH agar for cefoxitin DD was showed to result in an unacceptable number of VMEs relative to *mecA* PCR results (Miller et al., 2017). Therefore, testing on media other than unsupplemented MH agar for cefoxitin DD is discouraged by the CLSI (CLSI, 2020). Instead, PBP2a or *mecA* PCR tests are recommended (CLSI, 2020). Our data showed that reliable result may also be obtained by MIC testing with cation-adjusted Muller-Hinton broth. However, MIC testing after failed testing on MH agar would mean a longer turnaround time.

**FIGURE 4:**
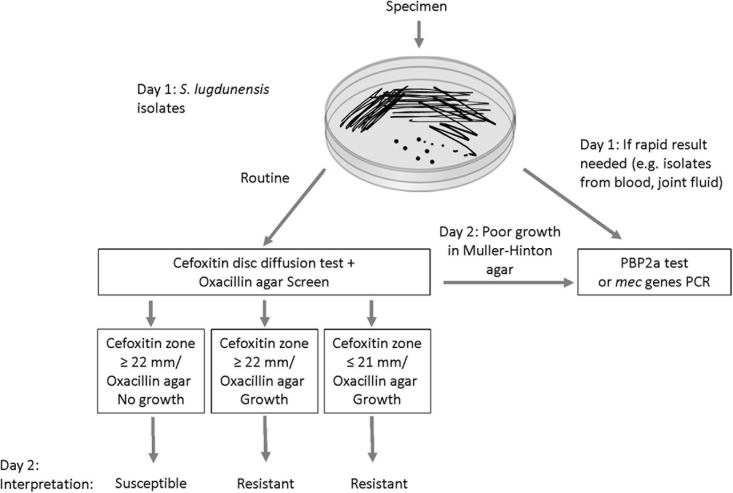
Proposed algorithm for improving the detection of *mecA*-mediated oxacillin resistance in *Staphylococcus lugdunensis.*

In laboratories that use DD testing as the primary method for susceptibility testing, we propose a new algorithm that includes the combined use of the oxacillin agar screen and cefoxitin disc as first line tests for *S. lugdunensis* (Figure 4). This will allow reliable detection of *mecA*-mediated resistance within the same timeframe as well as an assessment of the prevalence of oxacillin heteroresistance in this species. Isolates with cefoxitin zone ≤ 21 mm are interpreted as oxacillin-resistant. In our evaluation, all cefoxitin disc resistant isolates demonstrated growth in oxacillin agar screen (i.e., *mecA*-positive and homogeneous resistance). Isolates with growth in oxacillin agar screen but the cefoxitin zone is ≥ 22 mm should also be interpreted as resistant (i.e., *mecA*-positive and heteroresistance). Oxacillin-susceptible isolates are those with cefoxitin zone ≥ 22 mm and no growth in oxacillin agar screen (i.e., *mecA*-negative). We did not encounter any isolate with cefoxitin zone ≤ 21 mm with no growth in the oxacillin agar screen. If this occurs, such rare isolates can be further investigated using MIC, PBP2a or *mecA* PCR tests. In the present evaluation, ∼3% of the *S. lugdunensis* isolates grew poorly or failed to grow on MH agar. These isolates can be tested using MIC, PBP2a or *mec* genes PCR tests (Figure 4). *Staphylococcus lugdunensis* is a virulent pathogen. In order to inform timely treatment of patients with serious infections, *S. lugdunensis* colonies from positive blood cultures, joint fluid or other sterile sites can be directly tested using PBP2a or *mecA* PCR tests for rapid detection of oxacillin resistance (Figure 4).

This study is limited by the inclusion of isolates from a small geographic area. Since all of the isolates that gave VME results were of the same ST27 lineage, the VME rates may not be directly applicable to clinical isolates in other geographical areas. In Taiwan, some ST3 and ST27 *mecA*-positive isolates have also been found to be cefoxitin DD-susceptible (Kao et al., 2021). Inclusion of consecutively collected clinical isolates and a relatively large number of *mecA*-positive isolates are the present study’s strengths. As in *S. aureus*, studies have demonstrated the dissemination of *mecA* in *S. lugdunensis* is predominantly clonal (Ho et al., 2015; Yeh et al., 2016). In previous studies, the frequencies of ST27 among *S. lugdunensis* collections were variable, ranging from < 1 to 25% (Yeh et al., 2016; Dahyot et al., 2018; Noguchi et al., 2018). In our locality, this and our previous studies have demonstrated the widespread occurrence of the ST27/SCC*mec*V lineage in different patient populations (Ho et al., 2015, 2020). Further studies are required to investigate the international occurrence the ST27/SCC*mec*V lineage and the prevalence of false-susceptibility to oxacillin from heteroresistance in *S. lugdunensis*.

In conclusion, oxacillin resistance in some *mecA*-positive *S. lugdunensis* isolates cannot be reliably detected by the current disc (cefoxitin) and MIC (cefoxitin and oxacillin) breakpoints. The inclusion of an agar screening supplemented with 2 μg/ml oxacillin agar and 2% sodium chloride using a high bacterial inoculum will enable laboratories to improve detection of *mecA*-positive *Staphylococcus lugdunensis* isolates giving false-susceptibility results in disc and MIC testing. This could help clinicians to optimize antimicrobial therapy for patients.

### Take Home Message

• MecA-mediated beta-lactam resistance is emerging in *Staphylococcus lugdunensis*.

• Oxacillin agar screen but not cefoxitin disc test allowed reliable detection of *mecA*-mediated beta-lactam resistance in *S. lugdunensis*.

• Oxacillin agar screen can be included as a first line test and isolates that grow poorly on Muller-Hinton agar can investigated further with PBP2a tests or *mecA* PCR assays.

## Data Availability Statement

The datasets presented in this study can be found in online repositories. The names of the repository/repositories and accession number(s) can be found below: www.ncbi.nlm.nih.gov/, PRJNA727671.

## Author Contributions

P-LH, K-HC, AK-LW, CW-ST, VC-CC, and T-LQ designed and coordinated the study. CW-ST, VC-CC, and T-LQ provided the bacterial isolates. Y-HL and K-HC performed the experiments. P-LH wrote the manuscript and other authors critically reviewed and corrected the manuscript. All authors contributed to the study and are in agreement with the content of the manuscript.

## Conflict of Interest

The authors declare that the research was conducted in the absence of any commercial or financial relationships that could be construed as a potential conflict of interest.

## Publisher’s Note

All claims expressed in this article are solely those of the authors and do not necessarily represent those of their affiliated organizations, or those of the publisher, the editors and the reviewers. Any product that may be evaluated in this article, or claim that may be made by its manufacturer, is not guaranteed or endorsed by the publisher.

## Footnotes

1
https://bigsdb.pasteur.fr/
