# Supplementary material for: Improved Detection of mecA-Mediated β-Lactam Resistance in Staphylococcus lugdunensis Using a New Oxacillin Salt Agar Screen
Source: Front Microbiol. 2021 Aug 6;12:704552. doi: 10.3389/fmicb.2021.704552 (PMC8378274; doi:10.3389/fmicb.2021.704552)
Supplement: Supplementary file 1 [file Data_Sheet_1.pdf]

Supplementary file

Table S1. Number of isolates by patient location, specimen sources and *mecA* status

Patient location	Specimen source						No. of *mecA*		*mecA* pos		Total
	Wound[a]	Urine	Blood	Joint	Others (nonsterile)		Neg	Pos	%		
Surgery	63				1		57	7	10.9		64
AED	63						56	7	11.1		63
Orthopedic	36		1	1	1		32	7	17.9		39
Medical	28	1			1		20	10	33.3		30
Pediatrics	23	3					17	9	34.6		26
Outpatient	23						19	4	17.4		23
Renal	17						12	5	29.4		17
O&G	15		1				10	6	37.5		16
Others	15	2	2		2		15	7	31.8		22
Total	284	6	4	1	5[b]		238	62	20.7		300

Abbreviations: AED, accidents and emergency department; O&G, obstetrics and gynaecology; MLST

[a] Including wound tissue (n=24), pus (n=104), aspirate (n=3) and wound swabs (n=153)

[b]Including sputum (n=3) and stool (n=2).

Figure S1. Flowchart of the study design

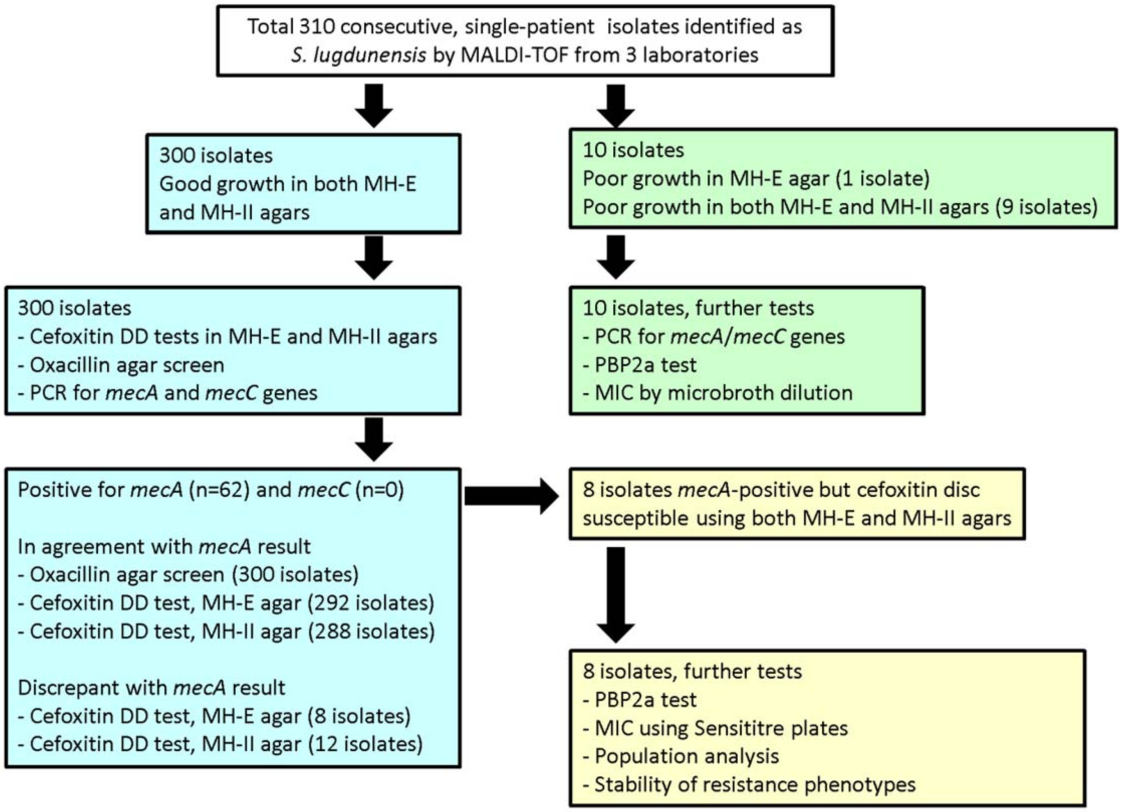

Abbreviations: DD, disc diffusion; MALDI-TOF, matrix-assisted laser desorption ionization time-of-flight; MH-II, Muller-Hinton-II; MH-E, Muller-Hinton-E
